# Dynamic Response of Sandwich Tubes with Continuously Density-Graded Aluminum Foam Cores under Internal Explosion Load

**DOI:** 10.3390/ma15196966

**Published:** 2022-10-07

**Authors:** Anshuai Wang, Xuehui Yu, Han Wang, Yu Li, Jie Zhang, Xueling Fan

**Affiliations:** 1 School of Mechatronic Engineering, Southwest Petroleum University, Chengdu 610500, China; 2 School of Science, Xi’an University of Architecture and Technology, Xi’an 710055, China; 3 Joint Research Center for Extreme Environment and Protection Technology, School of Aerospace Engineering, Xi’an Jiaotong University, Xi’an 710049, China

**Keywords:** blast resistance, density-graded foam, sandwich tubes, Voronoi technology, dynamic simulations

## Abstract

In this paper, the dynamic response of continually density-graded aluminum foam sandwich tubes under internal explosion load was studied. A 3D mesoscopic finite-element model of continually density-graded aluminum foam sandwich tubes was established by the 3D-Voronoi technology. The finite-element results were compared with the existing experimental results, and the rationality of the model was verified. The influences of the core density distribution, the core density gradient, and the core thickness on the blast resistance of the sandwich tubes were analyzed. The results showed that the blast resistance of the sandwich tube with the negative-gradient core is better than that of the sandwich tube with the uniform core. While the blast resistance of the sandwich tube with the positive-gradient core or the middle-hard-gradient core is worse than that of the sandwich tube with the uniform core. For the sandwich tube with the negative-gradient core, the core density gradient increased, and the blast resistance decreased. Increasing the thickness of the core can effectively decrease the deformation of the outer tube of the sandwich tube, but the specific energy absorption of both the whole sandwich tube and its core also decreases.

## 1. Introduction

Aluminum foam material has a long and almost constant plateau stress during compression. It can absorb a large amount of energy before being crushed to a stable stage or before failure, with excellent energy absorption and impact resistance, and has been widely used in explosion-proof and impact protection fields [1,2,3]. Compared with the single-foam material, the foam sandwich structure can show better anti-explosion performance under the explosion load [4,5,6,7,8,9].

A cylindrical shell is a common explosion-proof structure, widely used in protection against explosions. When the explosion load condition is fixed, the thickness of the sandwich structure panel, the relative density and thickness of the core, and the material parameters are the main parameters that affect its resistance to an explosion [10,11,12]. Karagiozova et al. [13] pointed out that when a certain quality is maintained, the anti-explosion performance of the sandwich tube with a relatively low foam core layer is better than that of the single-layer round tube and the deformation of the outer tube of the foam core sandwich tube with a relatively high density is larger than that of the single-layer round tube. Lu et al. [14] studied the impact resistance of aluminum-foam-filled pipes and traditional empty pipes through drop-weight impact experiments and numerical simulations. The results showed that the aluminum-foam-filled tube can avoid the sharp increase in impact force after compaction and exhibit better energy absorption characteristics.

The core of the sandwich structure can be designed well, introducing a gradient in the core further improves the mechanical properties of the sandwich structure, and the gradient core can exert the energy-absorbing properties of the core material better than the uniform core [15,16,17,18,19]. Lin et al. [20] studied the anti-explosion performance of different gradient cores through numerical simulation. The medium- and high-density gradient foam sandwich panels displayed the best blast resistance. Li et al. [21] conducted internal explosion load experiments and numerical simulations on foamed aluminum sandwich tubes. The results showed that the characteristic size of core cells has little effect on its energy absorption. For a uniform core, the energy absorption per unit mass decreases as the relative density increases. Zhang et al. [22] predicted the dynamic response of fully clamped double-layer rectangular foam sandwich panels and single-layer rectangular foam sandwich panels under explosion loads through analytical and numerical simulations and proposed the model solution for the large deflection of the double-layer sandwich panel. They found that when the quality of the material is the same, the impact resistance of the double-layer sandwich panel is better than that of the single-layer sandwich panel. Lin et al. [23] conducted low-speed impact experiments on gradient sandwich beams and carried out numerical simulations. The results showed that both the maximum contact force and the maximum deflection of the sandwich beam increase with an increase in the impact energy and the energy absorption rate of the core decreases with increasing impact energy.

Most commonly used finite-element models of foam materials, usually based on idealized solid element modeling, cannot describe the dynamic changes in the microstructure of porous materials [1,24]. Liang et al. [25,26] studied the dynamic response of and energy absorption by double-layer aluminum foam sandwich panels under explosion loads through experiments and 2D-Voronoi numerical simulations. The deformation analysis model of the positive- and negative-gradient sandwich cylinder under the action of an internal explosion load was established. The results showed that the positive-gradient foam specimen absorbs the maximum energy and transmits the most impulse to the back plate and the negative-gradient foam sample absorbs the least energy and transmits the least impulse to the back plate. Zhang et al. [27] applied 3D-Voronoi technology to generate closed-cell aluminum foam models. The compression characteristics and deformation mechanism of gradient aluminum foam under different impact velocities were studied by numerical simulation. On the basis of the 2D-Voronoi technology to build a hierarchical gradient aluminum foam sandwich tube and carry out numerical simulation, Liang et al. [28,29] conducted experimental studies on foamed aluminum sandwich tubes under internal explosion loads, and the results showed that the maximum deformation of the outer tube is related to the relative density and gradient of the foam core and the inner tube wall thickness has a great influence on the energy absorption of the sandwich tube structure.

The objective of this work was to study the dynamic response of sandwich tubes with continuous-density-gradient aluminum foam cores subjected to an explosion load. The paper is organized as follows. Section 2 presents the 3D-Voronoi modeling methods for the sandwich tube with a continuous-density-gradient aluminum foam core. In Section 3, the influence of the distribution mode of the core gradient, the relative density of the core, and the core thickness on the dynamic response of the sandwich tube under an internal explosion load are presented. Section 4 presents concluding remarks.

## 2. Finite-Element Simulation

### 2.1. Sandwich Tube Finite-Element Model

**Figure 1:**
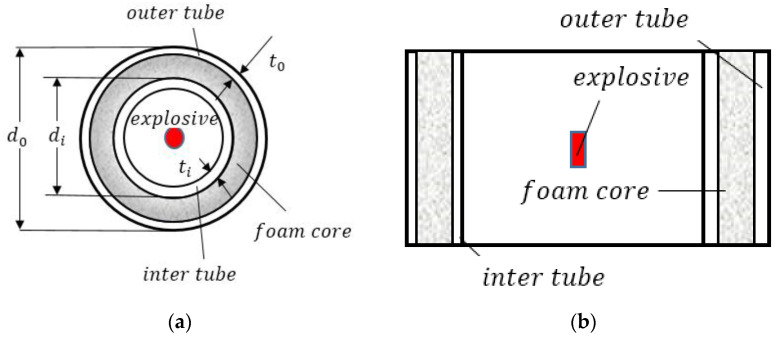
Schematic diagram of an aluminum foam sandwich tube. (**a**) Vertical view and (**b**) side view.

The aluminum foam sandwich tube structure consists of three parts: the aluminum foam core, the inner tube, and the outer tube. Figure 1 is a schematic diagram of the aluminum foam sandwich tube. The diameter of the outer tube of the structure is $d_{0}$ and the thickness is $t_{0}$. The diameter of the inner tube is $d_{i}$, and the thickness is $t_{i}$. The aluminum foam is filled between the inner and outer tubes. The aluminum foam core is of two types, uniform aluminum foam core and gradient aluminum foam core, in which the density of gradient aluminum foam core changes continuously along the thickness direction.

#### 2.1.1. Cylindrical 3D-Voronoi Foam Model

The 3D-Voronoi model is capable of describing the complex mesostructure of multicellular metallic materials. Therefore, it is widely used in the analysis of mechanical properties of cellular materials. The Voronoi structure is a Thiessen polygonal structure. In the plane, nucleation points are randomly generated and the vertical bisectors connecting adjacent points form a 2D-Voronoi structure. In 3D space, nucleation points are randomly generated and vertical bisectors connecting adjacent points form a 3D-Voronoi structure. To generate the 3D-Voronoi model, *N* nucleation points are randomly distributed in a space of volume *V*. The distance between adjacent nucleation points at different positions must satisfy:

(1) $$\delta_{ij}\geq \delta_{ij}^{min}=\left(1-k\right)\delta_{ij}^{0}$$

where $\delta_{ij}^{min}$ is the minimum distance between any two adjacent nucleation points and *k* is the irregularity defined by Zheng et al. [30] and *k* = 0.2. In this way, a set of gradient multicellular models with the same average density and a density distribution satisfying a linear relationship can be constructed. The relative density distribution of this model can be expressed as:

(2) $$\rho \left(x\right)=\rho_{0}\left[1+\gamma \left(z/H-1/2\right)\right]$$

**Figure 2:**
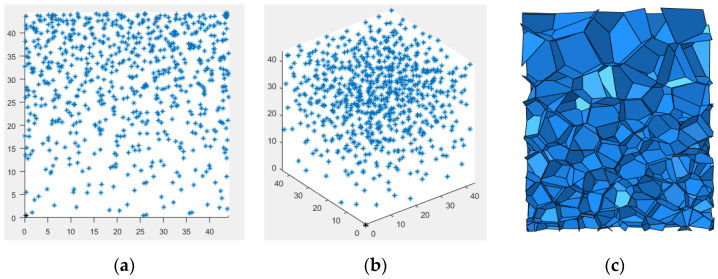
3D-Voronoi modeling process. (**a**) Front view of nucleation point, (**b**) oblique view of nucleation point, and (**c**) foam model.

The *z* direction is the direction of the density gradient, $\rho_{0}$ is the average relative density of the model, *γ* is the core density gradient of the model, and *H* is the length of the model density gradient direction. Figure 2 presents a schematic diagram of the nucleation point and its specific structural features.

Accordingly, two types of polar coordinate 3D-Voronoi foam models are constructed and the cartesian coordinates converted as follows:

(3) x=r×cosθy=r×sinθ

where *r* and *θ* are the radial and angular coordinates, respectively, in the polar coordinates of the nucleation point. Within a certain ring volume range, *N* nucleation points are randomly distributed and the distance between adjacent nucleation points at different positions satisfies $\delta_{ij}\geq \delta_{ij}^{min}=\left(1-k\right)\delta_{ij}^{0}$. The relative density distribution of the first polar coordinate 3D-Voronoi model can be expressed as follows:

(4) $$\rho \left(r\right)=\rho_{0}\left[1+\gamma \left(\frac{r-r_{1}}{R-r_{1}}-\frac{1}{2}\right)\right]$$

**Figure 3:**
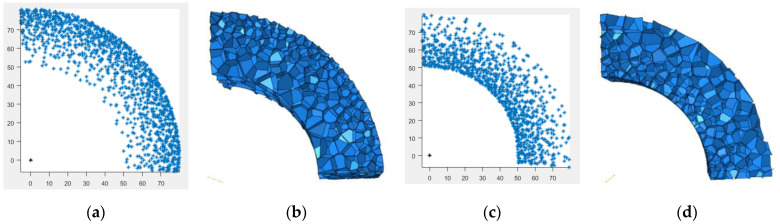
The first polar coordinate 3D-Voronoi modeling process. (**a**) Schematic diagram of P-type nucleation point, (**b**) P-type foam model, (**c**) schematic diagram of N-type nucleation point, and (**d**) N-type foam model.

In the formula, r is the direction of the density gradient, that is, the radial direction in polar coordinates; R is the outer diameter of the ring; and $r_{1}$ is the inner diameter of the ring. $R-r_{1}$ is the length of the model density gradient direction. According to the characteristics of the continuous gradient 3D-Voronoi model in the first polar coordinate, three different finite-element models were established: the positive-gradient core (P-type) sandwich tube, uniform core (U-type) sandwich tube and negative-gradient core (N-type) sandwich tube. The P-type sandwich tube refers to a tube with low density near the inner tube and high density far from the inner tube, and the N-type sandwich tube is the opposite. The specific structural features are shown in Figure 3.

The relative density distribution of the second cylindrical 3D-Voronoi model can be expressed as

(5) $$\rho \left(r\right)=\rho_{0}\left[1+\gamma \left(\frac{1}{2}-\left|1-\frac{2\left(r-r_{1}\right)}{R-r_{1}}\right|\right)\right]$$

**Figure 4:**
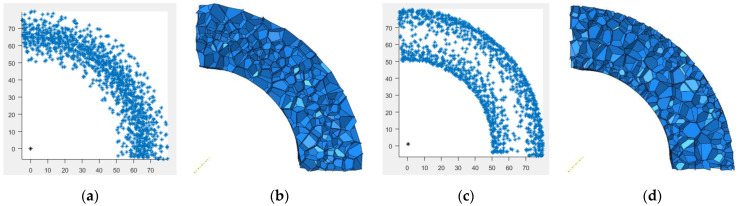
The second polar coordinate 3D-Voronoi modeling process. (**a**) Schematic diagram of MH-type nucleation point, (**b**) MH-type foam model, (**c**) schematic diagram of MS-type nucleation point, and (**d**) MS-type foam model.

The second type of continuous gradient 3D-Voronoi model in polar coordinates establishes the middle-hard-gradient core (MH-type) sandwich tube and the middle-soft-gradient core (MS-type) sandwich tube according to its density characteristics. The MH-type sandwich tube has high density in the middle and low density on both sides, and the MS-type sandwich tube is the opposite. The specific structural features are shown in Figure 4.

**Figure 5:**
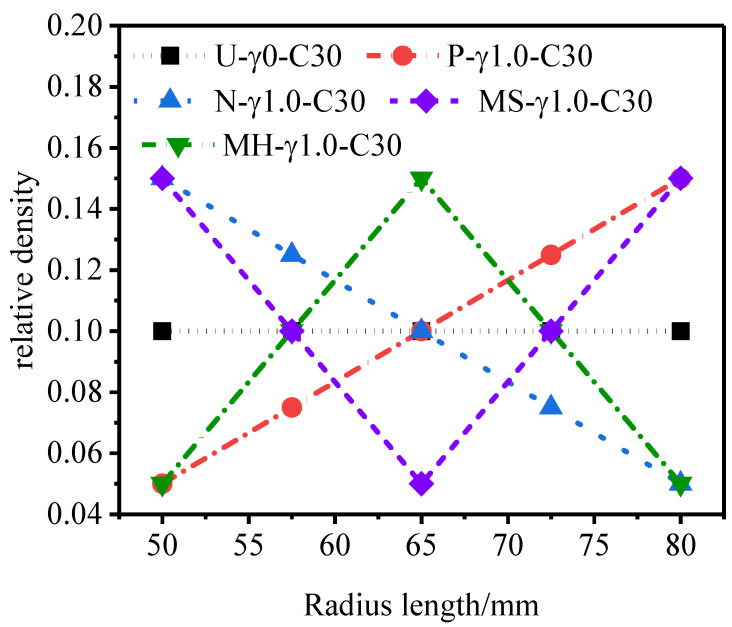
Density features of the 3D-Voronoi model.

Figure 5 presents the density characteristics of five continuous gradient 3D-Voronoi models in polar coordinates when the gradient *γ* = ±1.

#### 2.1.2. Meso Parameter Statistics

The number of cells in the gradient honeycomb can be estimated by the geometric characteristics. Within a finite length Δr in the gradient direction of the polar gradient honeycomb, the total length of the cell wall can be approximated as $\rho_{0}\omega r\Delta r/h_{0}$, where $h_{0}$ is the thickness of the cell wall and ω is the radian in the polar coordinate system. The number of cells in this finite region can be estimated as [30]

(6) $$\Delta n=\frac{\rho \left(r\right)\omega rH\Delta r/h_{0}}{6\left(1+2\sqrt{3}\right)l^{2}/2}$$

From the geometric characteristics of the honeycomb

(7) $$l=\frac{3\left(1+2\sqrt{3}\right)h_{0}}{8\sqrt{2}\rho }$$

where l is the average cell wall length in this finite region. Therefore, the total number of gradient honeycomb cells of P-γ type and N-γ type in polar coordinates can be given by the following formula:

(8) $$N=\frac{128\omega H\rho_{0}{}^{3}}{{\left(3\left(1+2\sqrt{3}\right)h_{0}\right)}^{3}}\int_{0}^{R}{\left[1+\gamma \left(\frac{r-r_{1}}{R-r_{1}}-\frac{1}{2}\right)\right]}^{3}r\;dr$$

Similarly, the total number of gradient honeycomb cells of MH-T type and MS-T type in polar coordinates can be given by the formula:

(9) $$N=\frac{128\omega H\rho_{0}{}^{3}}{{\left(3\left(1+2\sqrt{3}\right)h_{0}\right)}^{3}}\int_{0}^{R}{\left[1+\gamma \left(\frac{1}{2}-\left|1-\frac{2\left(r-r_{1}\right)}{R-r_{1}}\right|\right)\right]}^{3}r\;dr$$

#### 2.1.3. Finite-Element Model of the Sandwich Tube

**Figure 6:**
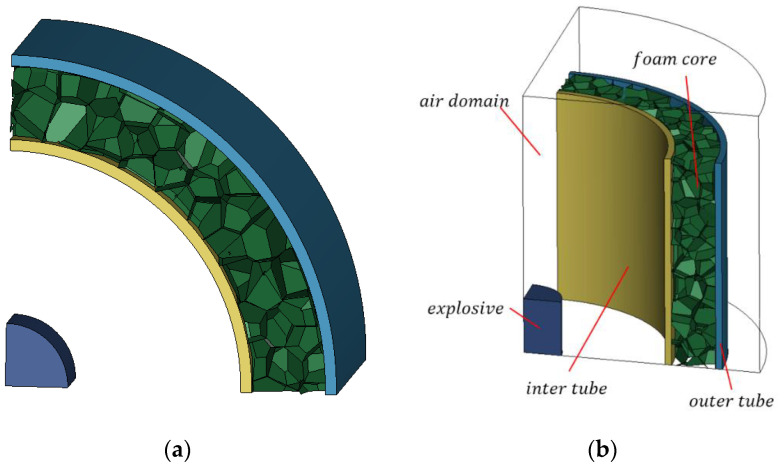
Computational model. (**a**) Schematic diagram of a foamed aluminum sandwich tube and (**b**) the finite-element model.

The finite-element software LS-DYNA was used to numerically simulate the response of the aluminum foam sandwich tube structure under the action of an internal explosion load. The air, explosives, and the inner and outer tubes used 8-node Solid164 solid elements, and the aluminum foam core adopted S3R and S4R shell elements. Figure 6 displays the established finite-element model. The contact between the inner tube, the outer tube, and the foamed aluminum core was AUTOMATIC_SURFACE_TO_SURFACE, and the contact of the aluminum foam core was AUTOMATIC_SINGLE_SURFACE. The friction coefficient of all contacts was set as 0.02 [29]. The explosive material was filled into the air by the initial volume fraction method, and the fluid–structure coupling algorithm was used between the aluminum foam sandwich tube and the air and the explosive. The arbitrary Lagrange Eulerian (ALE) method was used here. The method was explicit calculation. Because of the symmetry of the aluminum foam sandwich tube and the explosion load, an eighth model was established to reduce the calculation amount and symmetrical boundary conditions are imposed on the three sections of the aluminum foam sandwich tube. For the air model as a coupled domain, symmetric boundary conditions were imposed on three symmetry planes, and the remaining planes are defined as non-reflection boundaries to simulate the explosion of explosives in wireless air. The aluminum foam sandwich tube was divided into Lagrange meshes, and the air model was divided into Euler meshes. The total time was 500 μs and the scale factor for calculating the time step was selected as 0.67.

### 2.2. Material Properties

The material of the inner and outer circular tubes of the sandwich tube is made out of 45 steel. Considering the influence of the strain rate effect, the constitutive model adopted the Johnson–Cook model. Table 1 shows the material parameters of the Johnson–Cook model [28]. Because the aluminum foam is not sensitive to the strain rate effect, a simple model can be used; the bilinear elastic–plastic model was adopted as the matrix material: the density is 2730 $\mathrm{kg}/m^{3}$*,* the Young’s modulus is 70 GPa, Poisson’s ratio is 0.3, and the yield strength is 190 MPa [31,32,33,34]. The density of air is 1.293 $\mathrm{kg}/m^{3}$, the MAT_NULL constitutive model was adopted, the pressure cutoff of air is −1.000 × 10^−12^ [28], the state equation matching the constitutive model adopted EOS_LINEAR_POLYNOMIAL, and the pressure *P* in the equation of state is defined as a function of the internal energy density *e* and the relative volume *v*:

(10) $$P=C_{0}+C_{1}\mu +C_{2}\mu^{2}+C_{3}\mu^{3}+\left(C_{4}+C_{5}\mu +C_{6}\mu^{2}\right)e\;\mu =\frac{1}{v}-1$$

In the formula, $C_{0}$, $C_{1}$, $C_{2}$, $C_{3}$, $C_{4}$, $C_{5}$, and $C_{6}$ are material constants (take $C_{0}$ = $C_{1}$ = $C_{2}$ = $C_{3}$ = $C_{6}$ = 0 and $C_{4}$ = $C_{5}$ = 0.4); the initial internal energy density $e_{0}$ = 2.5 × 10^5^ J/m^3^; and the initial relative volume $v_{0}$ = 1.

The Johnson–Cook (J-C) model was applied to account for the strain-rate effects of the tubes. The J-C model is given as

(11) $$\sigma =\left[A+B\varepsilon^{n}\right]\left[1+c\ln\left({\dot{\varepsilon }}^{*}\right)][1-{\left(T^{*}\right)}^{m}\right]$$

where *ε* is the plastic strain of the material, $\dot{\varepsilon }*$ is the dimensionless strain rate of the material, $T^{*}$ is the homologous temperature defined as (T−$T_{room}$)/($T_{melt}$−$T_{room}$) where $T_{room}$ and $T_{melt}$ are the room and melting temperature, respectively. A is the quasi-static yield stress of the metal, B and n are the strain hardening coefficients, c is the strain rate hardening coefficient, and m is the thermal softening coefficient. The J-C parameters are listed in Table 1.

The detonation process of explosives is numerically simulated by the JWL equation of state and expressed as follows:

(12) $$P=A\left(1-\frac{\omega }{R_{1}V}\right)e^{-R_{1}V}+B\left(1-\frac{\omega }{R_{2}V}\right)e^{-R_{2}V}+\frac{\omega E}{V}$$

In the formula, A, B, $R_{1}$, $R_{2}$, and ω are constants; *E* is the initial specific internal energy of the explosive, and *V* is the initial relative volume of the explosive per unit volume. Table 2 shows the material parameters of the explosive.

In the simulation analysis, a sandwich tube structure with a uniform foamed aluminum core and four continuous gradient foamed aluminum core was considered. The relative density of the foamed aluminum core is 10%, and the wall thickness of the inner and outer tubes is 1.5 mm. To study the influence of the core density distribution, the core density gradient, and thickness of the foamed aluminum core on its energy absorption effect, and the detailed parameters of the relevant samples were set as shown in Table 3 and Table 4. The length of all sandwich tube specimens is 80 mm, and the length–diameter ratio of explosives is 1.5:1.

### 2.3. Finite-Element Model Verification

#### 2.3.1. Mesh Sensitivity Verification

**Figure 7:**
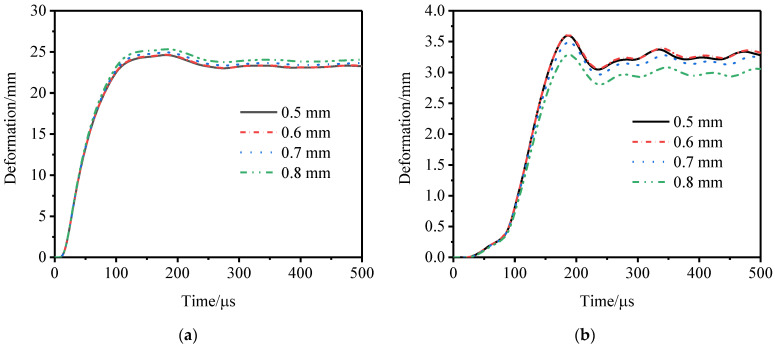
Deformation of inner and outer tubes with different mesh sizes. (**a**) Inner tube and (**b**) outer tube.

In a numerical simulation, a smaller number of grids will reduce the accuracy of the simulation and a larger number of grids will consume more computer resources. Therefore, it is necessary to find a grid size that ensures the accuracy of the simulation and consumes less computer resources. Figure 7 shows the time–history curves of the deformation of the inner and outer tubes of the specimen U-γ0-C30 under different grid sizes. When the grid size was less than 0.7 mm, a change in the grid size had little effect on the calculation results. When the grid size was 0.5 mm or 0.6 mm, the difference between the maximum deformation of the inner and outer tubes was extremely small and the maximum difference was 0.2%. Considering the computing resources and timeliness, the numerical simulation used the 0.6 mm grid.

#### 2.3.2. Comparison between Numerical Simulation and Experimental Results

**Figure 8:**
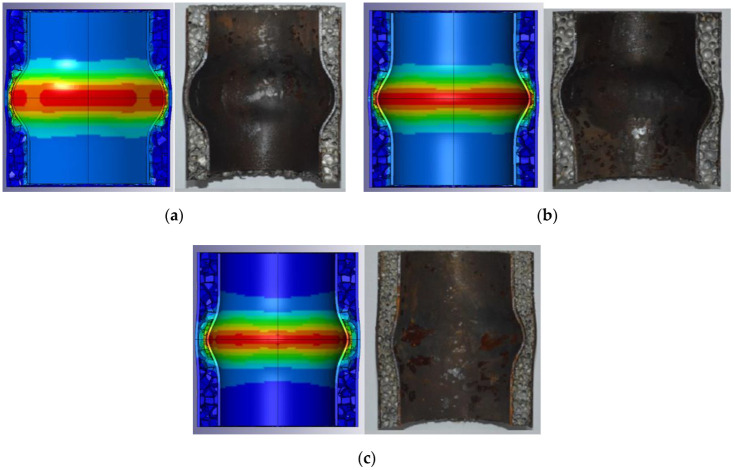
Comparison of numerical simulation and experimental results. (**a**) T1, (**b**) T2, and (**c**) T3.

To verify the correctness and rationality of the numerical model, it was compared with the three sets of experimental results (T1, T3, and T5) in the literature [28]. The sandwich tubes used in this experimental were produced with steel tubes and aluminum foam cores. The foam core was cut from 100 mm-thick foam panels by an electro-discharge machine to minimize the damage to the cell edges. The height of the tube was fixed at 100 mm. The thickness of tube was 1.5 mm. An aluminized explosive, JHL, was used in the blast experiments. The cylindrical explosive charge was held at the center of the sandwich tube using iron wires and detonated at its apex with a detonator. The length to radius ratio of the charge was equal to that of the internal tube. The sandwich tube was supported by plastic foams to reduce the influence of the reflected waves from the ground. The purpose of this setup is to minimize the end effects influence on the specimen. Each test was repeated twice. Figure 8 compares the numerical simulation and experimental results. Table 5 displays the geometric parameters of the T1, T3, and T5 specimens. The wall thickness of the inner and outer tubes is 1.5 mm. Table 5 shows the experimental results and numerical simulation results of the maximum deformation of the inner and outer tubes of the specimen. The numerical simulation results of the specimen are in good agreement with the experimental results, verifying the rationality and feasibility of the finite-element model.

## 3. Results and Discussion

The maximum deformation of the inner and outer tubes of the sandwich tube’s structure and energy absorption are important indicators for evaluating the anti-explosion performance of the sandwich tube structure. The sandwich tube structure should not only have good energy absorption characteristics but also be lightweight. Therefore, the maximum deformation of the inner and outer tubes is standardized:

(13) $$D_{s}=\frac{\delta_{T}}{m_{T}r_{T}}$$

where $\delta_{T}$ is the deformation of the inner tube/outer tube, $m_{T}$ is the mass of the inner tube/outer tube, and $r_{T}$ is the radius of the inner tube/outer tube.

The specific energy absorption $E_{sa}$ is defined as the energy absorbed per unit mass of the structure and is given by:

(14) $$E_{sa}=E_{a}/M$$

where $E_{a}$ is the energy absorbed by the structure and M is the mass of the structure.

### 3.1. Deformation Process

**Figure 9:**
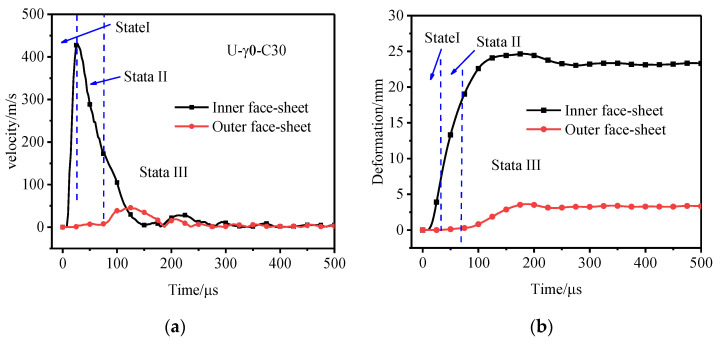
Deformation process of the aluminum foam sandwich tube. (**a**) Velocity–time curve of inner and outer tubes, and (**b**) deformation curve of inner and outer tubes.

Figure 9 shows the velocity curves and displacement curves of the inner and outer tubes of the foamed aluminum tube U-γ0-C30 under the explosion load. The deformation process of the foamed aluminum sandwich tube can be divided into three stages. In the first stage, the explosive inside the sandwich tube explodes, the shock wave spreads rapidly in the air and interacts with the inner tube, and the inner tube is accelerated to a high speed in an extreme time. At 27.5 μs, the velocity of the inner tube reaches a maximum value of 430 m/s. The inner tube squeezes the foam core in the process of accelerated deformation, but due to the extremely short time, the foam core is less compacted. In the second stage, the deformation rate of the inner tube gradually decreases and the foam core is further compacted and at the end of the second stage, the outer tube begins to deform. The following steps occur in the third stage: (i) the foam core layer is compacted, (ii) the speed and deformation of the outer tube begin to rise rapidly, (iii) the foam core is completely compacted, (iv) the speed of the outer tube rises briefly, (v) outer tube deformation reaches the maximum value, and (vi) outer tube deformation begins decrease. With the passage of time, the deformation values of the inner and outer tubes tend to stabilize.

### 3.2. Influence of the Core Density Distribution

**Figure 10:**
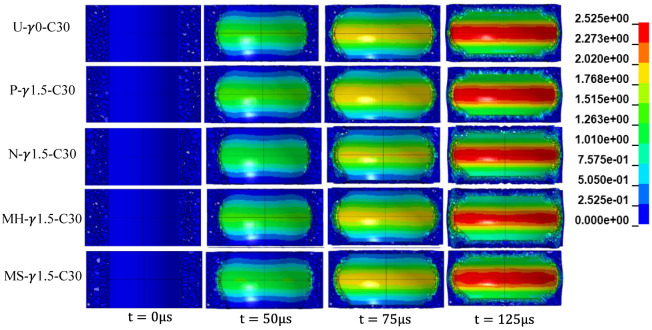
Cloud diagram of the deformation process of the foamed aluminum sandwich tube.

Figure 10 shows the deformation patterns of five kinds of aluminum foam sandwich tubes at different times under internal explosion loads. In the P-type sandwich tube, under the action of the explosion load, the low-density foam near the inner tube is compacted first and then the high-density foam is compacted again. Under the action of the explosion wave, the following stages occur: (i) part of the high-density foam near the inner tube of the N-type sandwich tube is compacted and extruded to a certain extent, (ii) part of the low-density foam is compacted, and (iii) the high-density foam and the low-density foam are compacted at the same time. In the MS-type sandwich tube, the low-density foam close to the inner tube is compacted, the middle high-density foam is compacted again, and then a part of the low-density foam close to the outer tube is compacted, and the low-density foam close to the outer tube and the middle high-density foam are compacted at the same time. In the MS-type sandwich tube, the high-density foam near the inner tube is compacted first, part of the middle low-density foam is compacted again, then both are compacted at the same time, and finally the high-density foam near the outer tube is compacted.

At 75 μs, the compression of the inner foam core of the N-type sandwich tube was significantly smaller than that of the U-type and P-type sandwich tubes. The reason is that the foam of the N-type sandwich tube close to the inner tube has a relatively large relative density and a strong ability to resist deformation. The compression of the inner foam core layer of the P-type sandwich tube was significantly larger than that of the U-type sandwich tube, because the foam of the P-type sandwich tube close to the inner tube is relatively less dense and has weaker resistance to deformation. Similarly, the compression amount of the inner aluminum foam core of the MH-type and MS-type sandwich tubes was between that of P type and N type. At 125 μs, among the five types of sandwich tubes, the deformation of the outer tube of the N-type sandwich tube was the smallest.

**Figure 11:**
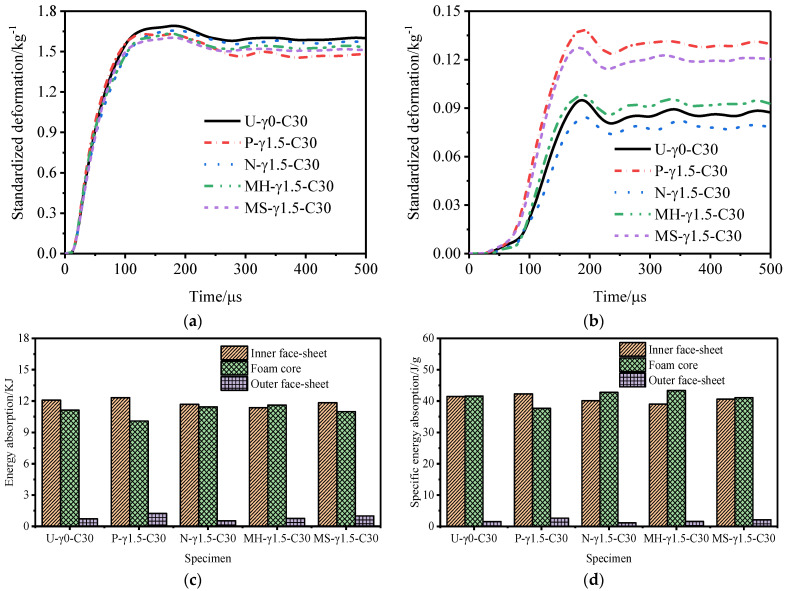
Anti−explosion performance of a foamed aluminum sandwich tube when the core density gradient is 1.5. (**a**) Deformation curve of the inner tube, (**b**) deformation curve of the outer tube, (**c**) total energy absorption of the sandwich tube, and (**d**) specific energy absorption of the sandwich tube.

**Figure 12:**
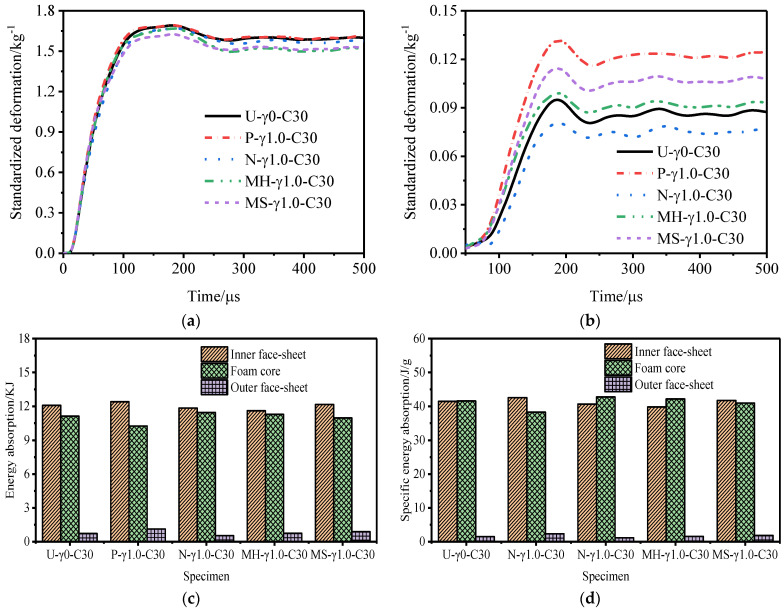
Anti−explosion performance of a foamed aluminum sandwich tube when the core density gradient is 1.0. (**a**) Deformation curve of the inner tube, (**b**) deformation curve of the outer tube, (**c**) total energy absorption of the sandwich tube, and (**d**) specific energy absorption of the sandwich tube.

**Figure 13:**
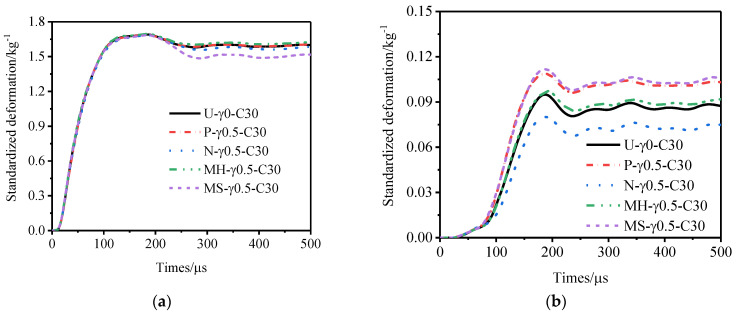

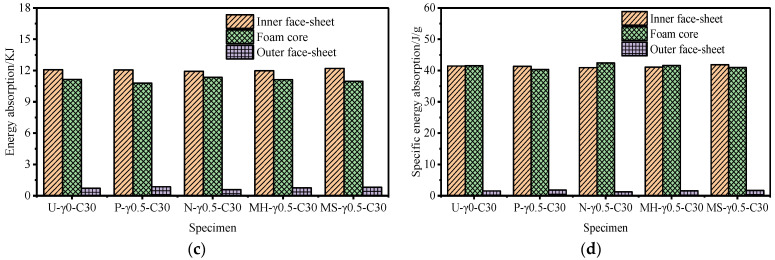
Anti−explosion performance of a foamed aluminum sandwich tube when the core density gradient is 0.5. (**a**) Deformation curve of the inner tube, (**b**) deformation curve of the outer tube, (**c**) total energy absorption of the sandwich tube, and (**d**) specific energy absorption of the sandwich tube.

Figure 11, Figure 12 and Figure 13 show the variation law of the deformation of the inner and outer tubes of the gradient sandwich tube with time and the total energy absorption and specific energy absorption of each part of the sandwich tube. When the core density gradients were 1.5, 1.0, and 0.5, there was little difference in the deformation of the inner tubes of the five sandwich tube models under the same explosion load. When the core density gradients were 1.5 and 1.0, the deformation of the outer tubes from large to small was as follows: P-type > MS-type > MH-type > U-type > N-type. When the core density gradient was 0.5, the deformation of the outer tube from large to small was as follows: MS-type > P-type > MH-type > U-type > N-type. However, at this time, the deformation of the outer tubes of MS-type and P-type was basically the same. Under the three core density gradients, the deformation of the outer tubes of the N-type sandwich tube was the smallest (smaller than the deformation of the outer tube of the U-type sandwich tube) and the deformation of the outer tubes of the other three models was larger than that of the U- type sandwich tube. From the total energy absorption and specific energy absorption diagrams of each part of the sandwich tube, it can be seen that the total energy absorption of the five models was basically the same. The total energy and specific energy absorption of the cores of N-type and MH-type was much higher than that by the other three types, and the total energy and specific energy absorption of the inner tube and the outer tube was lower. When the core density gradient was 0.5, the deformation of the outer tube of the N-type sandwich tube reached the lowest level, being 16.7% lower than that of the U-type sandwich tube and 37.8% lower than that of the P-type sandwich tube. Considering the deformation of the outer tube and the specific energy absorption of the structure, the anti-knock performance of the N-type sandwich tube was the best.

### 3.3. Influence of the Core Density Gradient

**Figure 14:**
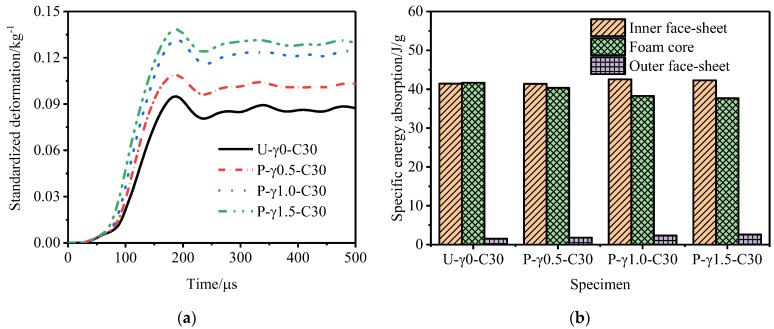
Anti−explosion performance of a P-type aluminum foam sandwich tube. (**a**) Deformation curve of the outer tube, and (**b**) specific energy absorption of sandwich tube.

**Figure 15:**
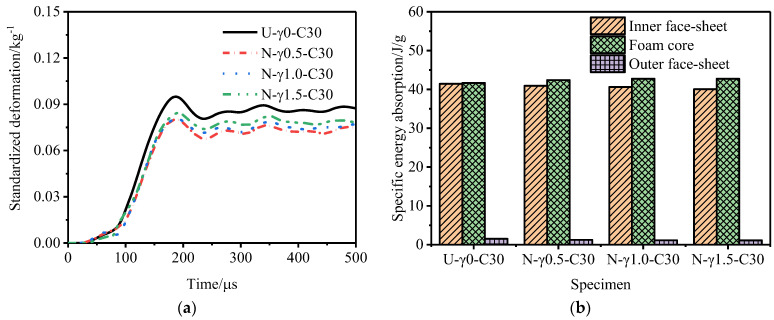
Anti−explosion performance of an N-type aluminum foam sandwich tube. (**a**) Deformation curve of the outer tube and (**b**) specific energy absorption of sandwich tube.

**Figure 16:**
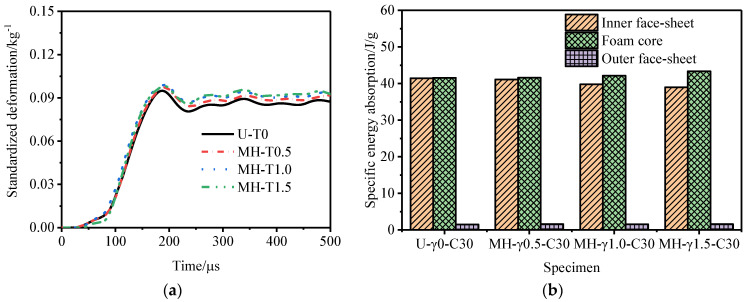
Anti−explosion performance of an MH-type aluminum foam sandwich tube. (**a**) Deformation curve of the outer tube and (**b**) specific energy absorbed of sandwich tube.

**Figure 17:**
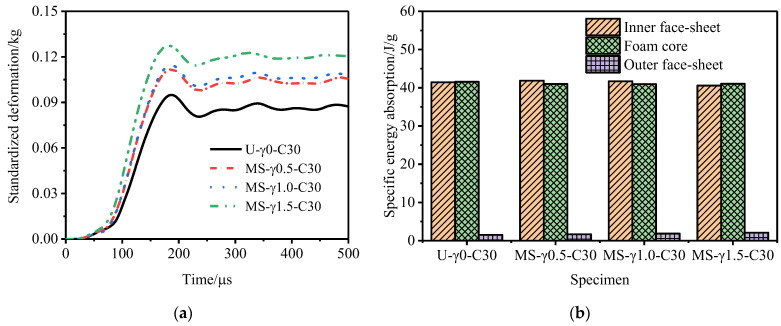
Anti−explosion performance of an MH-type aluminum foam sandwich tube. (**a**) Deformation curve of the outer tube and (**b**) specific energy absorbed by the sandwich tube.

Figure 14 shows the outer tube deformation of and specific energy absorption of the P-type sandwich tube under different core density gradients. With an increase in the core density gradient, the deformation of the outer tube of the P-type sandwich tube increased and the specific energy absorption of the inner tube and the outer tube increased, but the specific energy absorption of the core decreased. Considering the deformation of the outer tube and the specific energy absorption of the structure, the anti-explosion performance of the P-type sandwich tube was poor. Figure 15 shows the outer tube deformation of and the specific energy absorption of the N-type sandwich tube under different core density gradients. When the core density gradient of the N-type sandwich tube was 0.5, the deformation of the outer tube was the smallest, and as the gradient increased, the deformation of the outer tube increased and the specific energy absorption of the core increased slightly. Considering the deformation of the outer tube and the specific energy absorption of the structure, when the core density gradient was 0.5, the anti-knock performance of the N-type sandwich tube reached the optimum value. At this time, compared with the U-type sandwich tube, the deformation of the standardized outer tube was reduced by 16.7% and the specific energy absorption of the core was increased by 2.88%. Figure 16 shows the deformation of the outer tube and the specific energy absorption of the MH-type sandwich tube under different core density gradients. As the core density gradient increased, the deformation of the outer tube of the MH-type sandwich tube increased and the specific energy absorption of the core also increased. When the core density gradient was 1.5, the deformation of the normalized outer tube increased by 5.72% compared with that of the U-type sandwich tube, but the specific energy absorption of the core increased by 4.33%. Therefore, for the MH-type sandwich tube, the maximum deformation of the outer tube and the energy absorption are two contradictory evaluation indicators of anti-knock capability. Figure 17 shows the deformation of the outer tube and the specific energy absorption of the MS-type sandwich tube under different core density gradients. With an increase in the gradient, the deformation of the outer tube of the MS-type sandwich tube increased and the specific energy absorption of the core decreased. By and large, the anti-explosion performance of the MS-type sandwich tube was poor.

**Figure 18:**
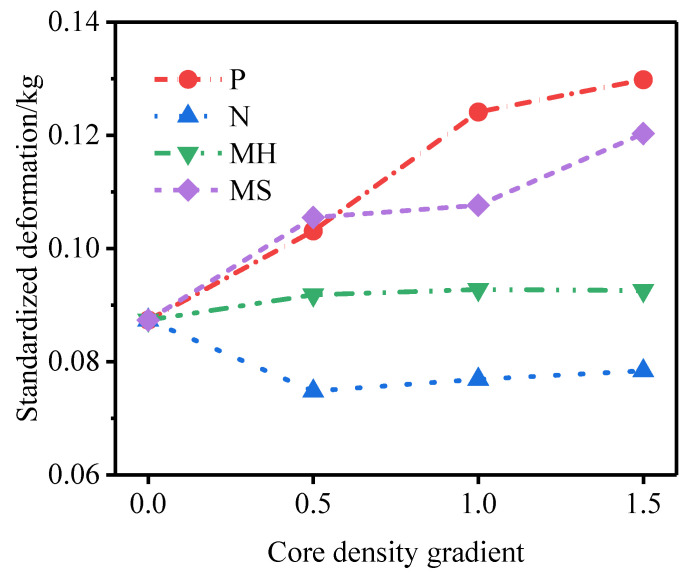
The influence of different core density gradients on the deformation of the outer tube.

**Figure 19:**
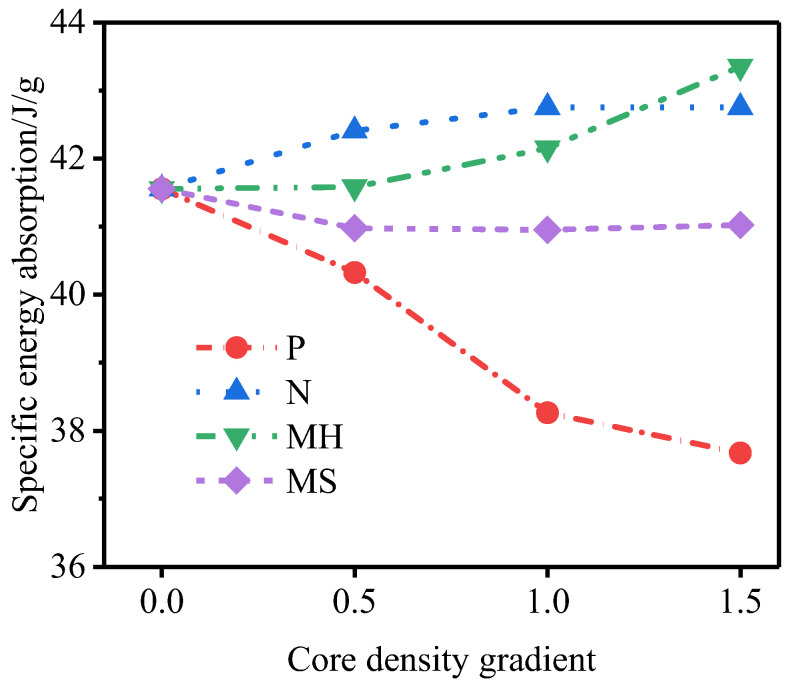
Specific energy absorption of foam core under different core density gradients.

Figure 18 shows the influence of different core density gradients on the deformation of the outer tube. Figure 19 shows the specific energy absorption of the foam core under different core density gradients. Under the three core density gradients, the deformation of the outer tube of the N-type sandwich tube and the specific energy absorption of the core were better than those of the U-type sandwich tube. When the core density gradient was 0.5, the anti-knock performance of the N-type sandwich tube reached the optimum value. Under the three core density gradients, the maximum deformation of and the energy absorption of the outer tube of the MH-type sandwich tube were two contradictory evaluation indicators of anti-knock capability. When the core density gradient was 1.5, the anti-knock performance of the MH-type sandwich tube reached the optimum value.

### 3.4. Influence of the Core Thickness

**Figure 20:**
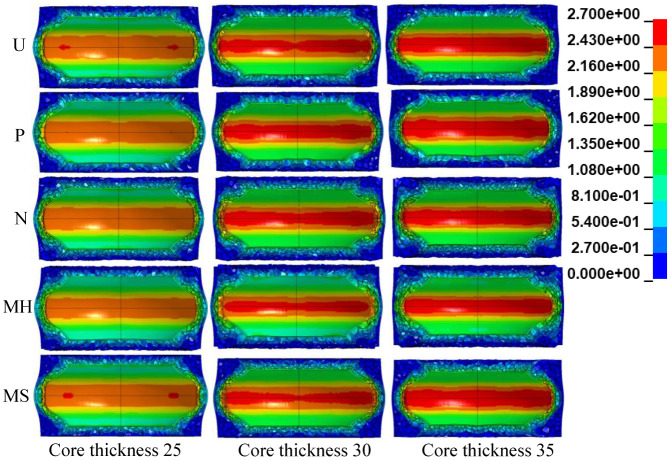
Deformation diagrams of sandwich tubes with different core thicknesses.

When the inner tube size was fixed and the core density gradient was 1.0, as the core thickness of the foam increased from 25 mm to 35 mm, the mass of the sandwich tube structure increased from 924.7 g to 1123.2 g, an increase of 21.47%. The anti-knock performance had a great impact. Figure 20 shows the deformation diagrams of the five kinds of aluminum foam sandwich tubes with different core thicknesses under the internal explosion load. The specimens with thinner foam cores underwent a higher degree of crushing, and those with thicker cores were not completely crushed. 

**Figure 21:**
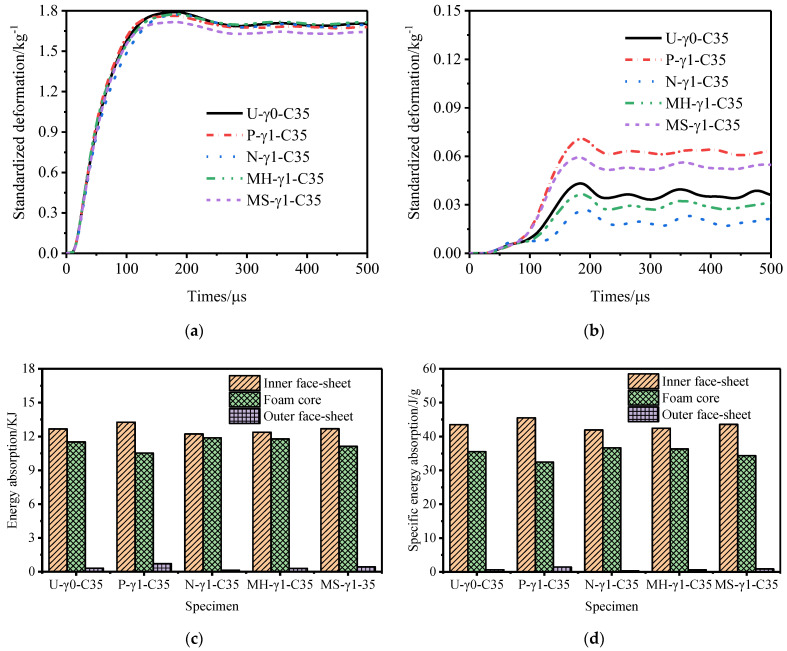
Anti−explosion performance of a foamed aluminum sandwich tube when the core thickness is 35 mm. (**a**) Deformation curve of the inner tube, (**b**) deformation curve of the outer tube, (**c**) total energy absorption of the sandwich tube, and (**d**) specific energy absorption of the sandwich tube.

**Figure 22:**
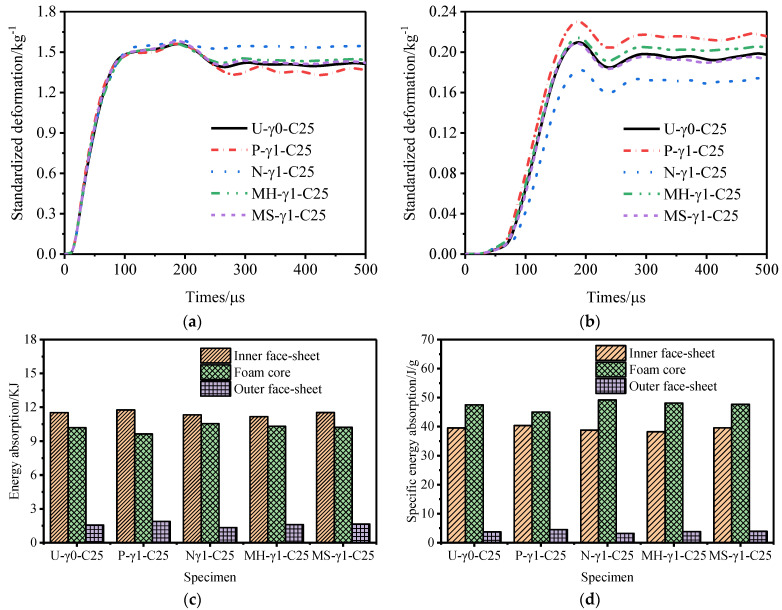
Anti−explosion performance of a foamed aluminum sandwich tube when the core thickness is 25 mm. (**a**) Deformation curve of the inner tube, (**b**) deformation curve of the outer tube, (**c**) total energy absorption of the sandwich tube, and (**d**) specific energy absorption of the sandwich tube.

**Figure 23:**
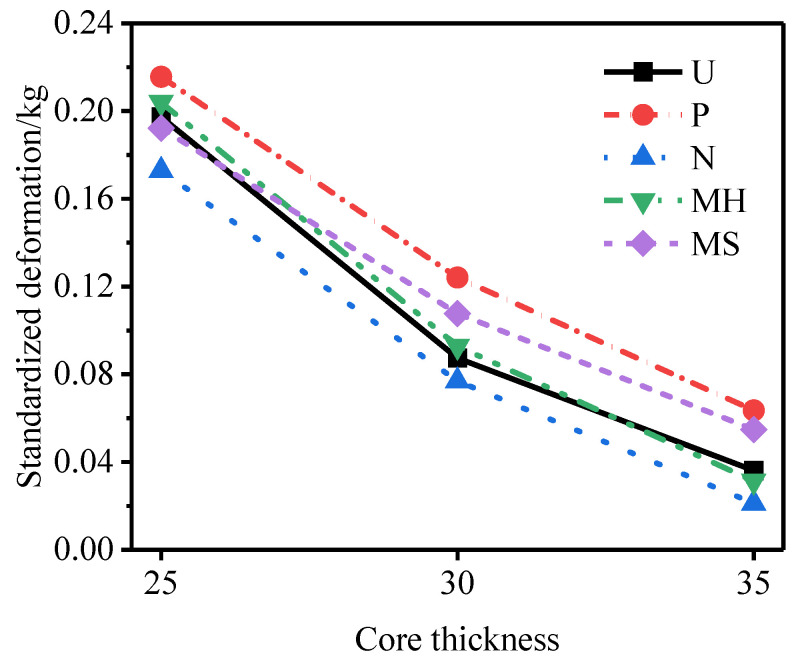
Deformation of the outer tubes of sandwich tubes with different core thicknesses.

Figure 21 and Figure 22 show the deformation curves of the inner and outer tubes of the sandwich tube and the total energy absorption and specific energy absorption of the sandwich tube structure when the core thicknesses were 35 mm and 25 mm. When the core thickness was 30 mm, the deformation curves of the inner and outer tubes of the sandwich tube and the total energy absorption and specific energy absorption of the sandwich tube structure are shown in Figure 14. With an increase in the core thickness, the deformation of the inner tube of the five kinds of aluminum foam sandwich tubes gradually increased but the deformation of the outer tube significantly decreased. Because the N-type sandwich tube has the best anti-explosion performance, here is an example of the N-type sandwich tube. When the core thickness increased from 25 mm to 30 mm, the deformation of the inner tube increased by 2.58% but the deformation of the outer tube decreased by 55.65%. When the core thickness increased from 30 mm to 35 mm, the deformation of the inner tube increased by 7.12% but the deformation of the outer tube decreased by 73.02% (a significant decrease). Figure 23 shows the deformation of the outer tubes of sandwich tubes with different core thicknesses. 

**Figure 24:**
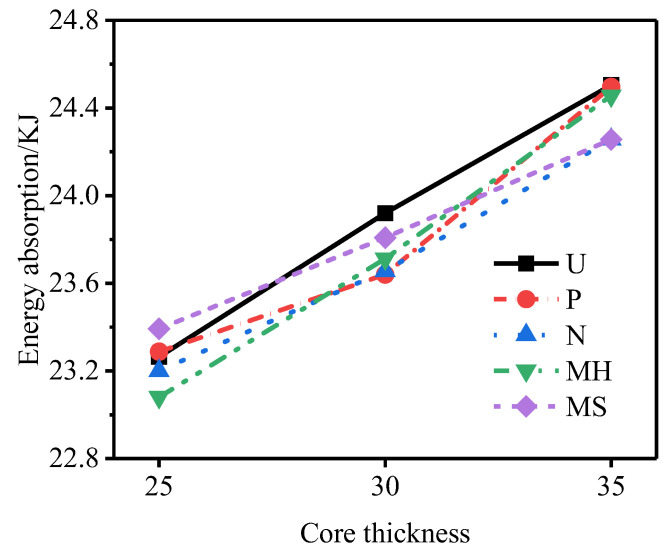
Total energy absorption of sandwich tubes with different core thicknesses.

**Figure 25:**
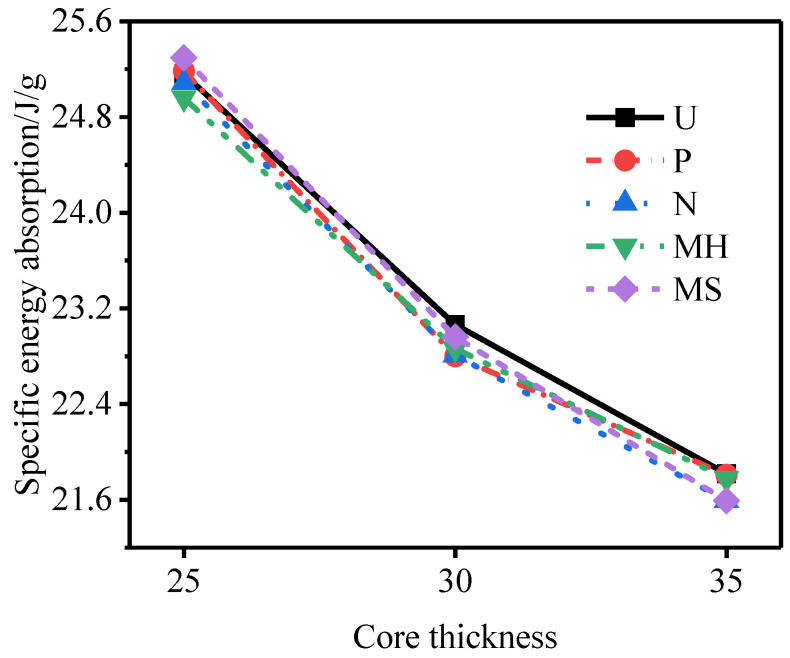
Total specific energy absorption of sandwich tubes with different core thicknesses.

**Figure 26:**
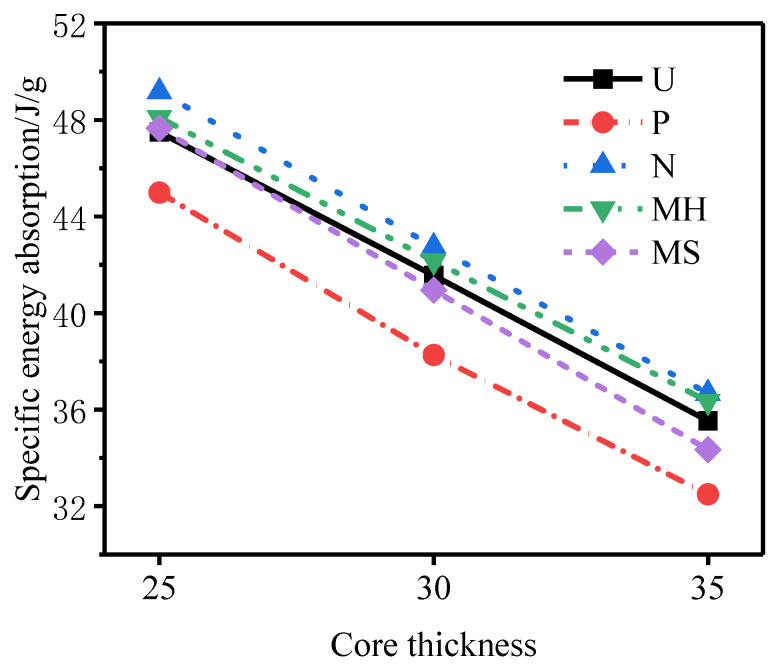
Core specific energy absorption of sandwich tubes with different core thicknesses.

Figure 24, Figure 25 and Figure 26 show the total energy absorption, the total specific energy absorption, and the core specific energy absorption of tubes of different core thicknesses. With an increase in the core thickness, the total energy absorption of each structure of the sandwich tube increases but the specific energy absorption decreases, because with the increase in the core thickness, the mass of the specimen also increases. When the core thickness increased from 25 mm to 30 mm, the total energy absorption of the sandwich tube increased by 1.96%, the total specific energy absorption decreased by 9.11%, and the core-specific energy absorption decreased by 13.15%. When the core thickness increased from 30 mm to 35 mm, the total energy absorption of the sandwich tube increased by 2.50%, the total specific energy absorption decreased by 5.41%, and the core-specific energy absorption decreased by 14.01%. In conclusion, increasing the thickness of the core can effectively reduce the deformation of the outer tube and improve the total energy absorption, but it will reduce the total specific energy absorption and the specific energy absorbed by the core.

## 4. Conclusions

Based on 3D-Voronoi technology, a finite-element model of a continuous gradient aluminum foam sandwich tube under polar coordinates was established. The simulation results were compared with the existing experimental results, and the rationality of the model was verified. On this basis, the dynamic response of the core tube under an internal explosion load was studied, and the influence of the core density distribution, the core density gradient, and the thickness of the core on the mechanical properties of the aluminum foam sandwich tube was analyzed. The main conclusions are as follows:

(1) When the core density gradient is the same, the deformation of the outer tube of the N-type sandwich tube and the specific energy absorption of the core are better than those of the U-type sandwich tube. The deformation of the outer tubes of P-type and MS-type and the specific energy absorption of the core are worse than those of the U-type sandwich tube. The deformation of the MH-type outer tube is worse than that of the U-type sandwich tube, but its core displays better specific energy absorption than that of the U-type sandwich tube.
(2) For N-type sandwich tubes, with decrease in the core density gradient, the deformation of the outer tube decreases but the specific energy absorption by the core is basically unchanged. For MH-type sandwich tubes, with an increase in the core density gradient, the deformation of the outer tube decreases and the specific energy absorption of the core increases. For P type and MS-type sandwich tube, with an increase in the core density gradient, the deformation of the outer tube increases and the specific energy absorption of the core decreases. Among the five gradient sandwich tubes, the N-type sandwich tube displays the best resistance to an explosion.
(3) Under the same explosive load, although increasing the core thickness will reduce the total specific energy absorption by the sandwich tube, it will effectively reduce the deformation of the outer tube and increase the total energy absorption. The degree of improvement of the deformation of the outer tube is far greater than the reduction in the specific energy absorption. Considering the deformation of the outer tube and the specific energy absorption of the core, the resistance of the N-type sandwich tube to an explosion is still the best for the three core thicknesses.

## Figures and Tables

**Table 1:** Material parameters of the Johnson–Cook model [28].

Material	Density/kg/m^3^	Young’s Modulus/GPa	*A*/MPa	*B*/MPa	*n*	*c*	*m*
Steel	7850	210	507	320	0.28	0.064	1.06

**Table 2:** Material parameters of explosives [28].

Material	Density/ kg/m^3^	Detonation Velocity/m/s	*A*/GPa	*B*/GPa	*R*_1_	*R*_2_	*ω*	*E*/GJ/m^3^	*V*
JHL-3	1650	7050	611	10.7	4.4	1.2	0.35	8.9	1.0

**Table 3:** Geometric parameters of aluminum foam sandwich tubes with different gradients.

Specimen Number	Outer Tube Diameter *d*_0_/mm	Inner Tube Diameter *d_i_*/mm	Outer Tube Wall Thickness *t*_0_/mm	Inner Tube Wall Thickness *t_i_*/mm	Relative Density *ρ*/%	Specimen Quality *M*/g	Core Density Gradient
U-γ0-C30	163	100	1.5	1.5	10	1037	0
P-γ1.0-C30	163	100	1.5	1.5	10	1037	1
N-γ1.0-C30	163	100	1.5	1.5	10	1037	1
MH-γ1.0-C30	163	100	1.5	1.5	10	1037	1
MS-γ1.0-C30	163	100	1.5	1.5	10	1037	1
P-γ0.5-C30	163	100	1.5	1.5	10	1037	0.5
N-γ0.5-C30	163	100	1.5	1.5	10	1037	0.5
MH-γ0.5-C30	163	100	1.5	1.5	10	1037	0.5
MS-γ0.5-C30	163	100	1.5	1.5	10	1037	0.5
P-γ1.5-C30	163	100	1.5	1.5	10	1037	1.5
N-γ1.5-C30	163	100	1.5	1.5	10	1037	1.5
MH-γ1.5-C30	163	100	1.5	1.5	10	1037	1.5
MS-γ1.5-C30	163	100	1.5	1.5	10	1037	1.5

**Table 4:** Geometric parameters of aluminum foam sandwich tubes with different thicknesses.

Specimen Number	Outer Tube Diameter *d*_0_/mm	Inner Tube Diameter *d_i_*/mm	Outer Tube Wall Thickness *t*_0_/mm	Inner Tube Wall Thickness *t_i_*/mm	Relative Density *ρ*/%	Specimen Quality *M*/g	Core Density Gradient
U-γ0-C35	173	100	1.5	1.5	10	1123.2	0
P-γ1.0-C35	173	100	1.5	1.5	10	1123.2	1
N-γ1.0-C35	173	100	1.5	1.5	10	1123.2	1
MH-γ1.0-C35	173	100	1.5	1.5	10	1123.2	1
MS-γ1.0-C35	173	100	1.5	1.5	10	1123.2	1
U-γ0-C25	153	100	1.5	1.5	10	924.7	0
P-γ1.0-C25	153	100	1.5	1.5	10	924.7	1
N-γ1.0-C25	153	100	1.5	1.5	10	924.7	1
MH-γ1.0-C25	153	100	1.5	1.5	10	924.7	1
MS-γ1.0-C25	153	100	1.5	1.5	10	924.7	1

**Table 5:** Comparison of numerical simulation and experimental results.

Specimen Number	Inner Tube Diameter /mm	Outer Tube Diameter/mm	Specimen Length/mm	Relative Density	Numerical Simulation		Experimental Results		Relative Error	
					Inner Tube/mm	Outer Tube/mm	Inner Tube/mm	Outer Tube/mm	Inner Tube/ mm	Outer Tube/ mm
T1	67	90	100	11%	9.930	0.564	9.80	0.58	1.33%	2.76%
T3	67	90	100	11%	8.483	0.806	9.35	0.745	9.27%	8.19%
T5	67	90	100	11%	6.867	0.706	6.90	0.75	1.65%	5.87%

## Data Availability

The data presented in this study are available on reasonable request from the corresponding author.
